# The effect of cadmium on the growth and antioxidant response for freshwater algae *Chlorella vulgaris*

**DOI:** 10.1186/s40064-016-2963-1

**Published:** 2016-08-08

**Authors:** Jinfeng Cheng, Hongchen Qiu, Zhaoyang Chang, Zaimin Jiang, Wenke Yin

**Affiliations:** 1 The College of Life Sciences, Northwest A&F University, Yangling, 712100 Shaanxi China; 2 College of Mechanical and Electronic Engineering, Northwest A&F University, Yangling, 712100 Shaanxi China

**Keywords:** *Chlorella vulgaris*, Cadmium, Reactive oxygen species (ROS), Antioxidant enzymes

## Abstract

The objective of the present work was to evaluate the effect of exogenously applied cadmium on the physiological response of green algae *Chlorella vulgaris*. The study investigated the long-term effect (18 days) of cadmium on the levels of algae biomass, assimilation pigment composition, soluble protein, oxidative status (production of hydrogen peroxide and superoxide anion), antioxidant enzymes (such as superoxide dismutase, peroxidase, catalase and glutathione reductase enzyme) in *C. vulgaris*. The results showed that growth, the amount of chlorophyll *a* (*Chl a*), chlorophyll *b* (*Chl b*) and carotenoids gradually decreased with increasing cadmium over 18 days exposure. Cadmium at concentration of 7 mg L^−1^ inhibited algal growth expressed as the number of cells. Our research found that *C. vulgaris* has a high tolerance to cadmium. Contents of chlorophylls (*Chl a* and *Chl b*) and carotenoids (Car) of *C. vulgaris* was significantly decline with rising concentration of cadmium (p < 0.05). The decrease of 54.04 and 93.37 % in *Chl a*, 60.65 and 74.32 % in *Chl b*, 50.00 and 71.88 % in total carotenoids was noticed following the treatment with 3 and 7 mg L^−1^ cadmium doses compared with control treatment, respectively. Cadmium treatments caused a significant change in the physiological competence (calculated as chlorophyll *a*/*b*) which increased with increasing Cd(II) doses up to 1 mg L^−1^ but decreased at 3 mg L^−1^. While accumulation of soluble protein was enhanced by presence of cadmium, the treatment with cadmium at 3 and 7 mg L^−1^ increased the concentration of soluble proteins by 88, 95.8 % in *C. vulgaris*, respectively. Moreover, low doses of cadmium stimulated enzymatic (superoxide dismutase, catalase and glutathione reductase) in *C. vulgaris*, The content of peroxidase increased with the increasing cadmium concentration, and had slightly decreased at the concentration of 7 mg L^−1^, but was still higher than control group, which showed that cadmium stress at high concentration mainly peroxidase works in *C*. *vulgaris*. And therefore, suppressed reactive oxygen species (hydrogen peroxide and superoxide) accumulated. The present study also showed that cadmium increased oxidative stress and induced antioxidant defense systems against reactive oxygen species. The observation in here analyzed *C. vulgaris* after exposure to cadmium indicate that hydrogen peroxide, superoxide and peroxidase in the alga with exposure to Cd(II) seemed to be parameters as biomarkers for metal-induced oxidative stress.

## Background

Heavy metals are important environmental pollutants, water environmental deterioration caused by heavy metal emissions are increasing, and produce toxic effects on aquatic plants. The bioaccumulation of metals is a useful indicator since metals are not metabolized (Luoma and Rainbow 2005). Heavy metal can accumulate in aquatic animals and aquatic plants, not only effect the growth and development of aquatic animals and aquatic plants seriously, but also may enter the food chain and endanger human health. Algae are the primary producers of water, heavy metals and other pollutants which into the water through various means are first act on the algae, algae-rich contaminants and affect the entire aquatic ecosystem by pass through the food chain (Liu et al. 2008). Recent studies show that algae can enrich metal ions, we can use algae to repair heavy metal contaminated water. Study the algae physiological response mechanism in heavily stress conditions, to explore the mechanism of resistance to heavy metals, can provide a reference for better used in the treatment of heavy metal wastewater. Under normal metabolic process, the enzymatic and non-enzymatic protection system can make the production and eliminate of ROS maintain homeostasis. When subjected to environmental stress, such as, strong light (Romanowska et al. 2008), ultraviolet radiation (Zhang et al. 2005; Schmidt et al. 2011) and heavy metal stress (Dai 2012), will produce reactive oxygen species (ROS), such as superoxide (O_2_^−·^), hydroxide (OH^−^), hydroxyl radical (·OH), hydrogen peroxide (H_2_O_2_). Heavy metal has some toxicity; trace amounts of heavy metals can produce toxic effects. Stress leads to plants produce large amounts of reactive oxygen species. ROS can directly damage proteins, amino acids, nucleic acids and membrane lipids. The antioxidant protection system like the superoxide dismutase (SOD), peroxidase (POD), catalase (CAT), glutathione reductase enzyme (GR), et al can remove the excess ROS induced by stress, protecting cells against injury (Zhou et al. 2001). SOD is the first key enzyme to scavenging reactive oxygen species in plants; in organisms it is in an important position in the active oxygen metabolism, which can disproportionate O_2_^−^ to be H_2_O_2_, thereby protecting cells oxidative free radical damage. POD is also one of a plant antioxidant enzyme defense system, its active can reflect the intensity of antioxidant capacity and the severity of poisoned of plants, and can catalyze the decomposition the toxic substances in a certain range (Zhang et al. 2002). And POD able to restore H_2_O_2_ into H_2_O. CAT is also a ubiquitous enzyme that can remove H_2_O_2_ generated in the metabolism in plant. GR plays an important role in the glutathione cycle metabolism, plants can resistant oxidative metabolism by glutathione metabolic cycle. And under stress, the morphology, growth, photosynthetic pigments, cell biology and physiology of algal were also affected (Bouzon et al. 2011; Schmidt et al. 2011).

*Chlorella vulgaris* is Chlorophyceae, single-cell green freshwater algae, the diameter is 3–8 microns, is a highly efficient photosynthetic plants, and one of the earliest life on earth, is commonly found in freshwater ecosystems. Survival of the green algae in the aquatic environment contaminated with metals depends on its ability to generate and transit signals that adjust the metabolism. Biomarkers can be used to evaluate the ecological risk assessment (Çelekli et al. 2016). *Chlorella vulgaris* has strong ability to adapt to the environment. In our previous study of the chlorella, our research found that *C. vulgaris* has a high tolerance to cadmium. Cadmium is one of the most toxic metals. The major sources of cadmium release into the environment by waste streams are electroplating, smelting, alloy manufacturing, pigments, plastic, battery, mining, and refining processes (Gülay and Yakup 2011). But we found that the study on effect of physiological and antioxidant enzymes on *C. vulgaris* by cadmium is less.

In view of this, the present study was designed to investigate the extent of Cd-induced oxidative stress in *C. vulgaris*. The effect of various cadmium concentration on *C. vulgaris* growth, pigments, hydrogen peroxide (H_2_O_2_), superoxide anion (O_2_^·−^), SOD, POD, CAT and GR have been investigated. Provide evidence of physiological mechanisms in the aspects of response cadmium stress by plants. The effect of cadmium on algae growth and antioxidant system and the physiological of *C. vulgaris* response to cadmium stress were analyzed, it aimed to further explore the mechanism of metal toxicity to algae and the mechanism of resistance to heavy metals.

## Methods

Clonal culture of *C*. *vulgaris* was established by micropipette isolation of a single cell from the water sample which was collected from freshwater, Shaanxi Province, China. Cultures were grown under sterile conditions on glass triangular flask with BG11 medium (Stanier et al. 1971). Cultures were maintained at 20 °C under 12 h light: 12 h dark (L: D) cycle and at an illumination of 75 µmol photons m^−2^ s^−1^.

Cells were harvested by centrifugation at exponential phase, collected algae (which density was 3 × 10^4^ cells mL^−1^) were enriched in triplicate with varying Cd(II) supplements in the final concentrations of 0.0, 0.5, 1, 3, 5, 7 mg L^−1^. In all cases, 3CdSO_4_·8H_2_O was used.

### Determination of cell growth

Subsamples for cell counting (2 mL) and metal concentration were taken at approximately the same time every day. Samples for enumeration were fixed in Lugo’s solution (final concentration 2 %) and counted in Sedgewick rafter chamber. Biomass is represented by the number of algae (Lundholm et al. 2004).

### Measurement of pigments

The chlorophyll was extracted in the dark for 1 h at 65 °C in 5 mL DMSO. After cooling to room temperature and centrifuged at 15,000*g* for 15 min. The chlorophyll content was estimated according to the equations proposed by Wellburn (1994) using a spectrophotometer at 666, 653, and 750 nm to correct unspecific absorption (Jozef and Martin 2007). To determine the content of “total” carotenoids, absorbance was read at 480 nm. *Chl a*, *Chlb*, chlorophyll *a* + b and total carotenoids were calculated using equations derived from specific absorption coefficients for pure *Chla* and *Chlb* in DMSO (Wellburn 1994). Chlorophyll *a/b* was used to assess the physiological competence of algal cells.

### Measurement of soluble protein

Soluble protein was measured according to Coomassie Brilliant Blue G-250 method (Bradford 1976). Proteins were extracted with 50 mM potassium phosphate buffer (pH 7.0) and estimated using bovine serum albumin as standard. After centrifugation at 5000*g* at 4 °C for 10 min, the water-soluble protein content of supernatants was measured. Supernatants (1 mL) were added into 5 mL Coomassie Brilliant Blue G-250 and mixed thoroughly. After 10 min, absorbance of samples (2 mL) was spectrophotometrically measured at 595 nm. Each treatment was replicated three times.

### Detection of hydrogen peroxide and superoxide anion

Hydrogen peroxide was extracted by potassium phosphate buffer (pH 6.5).Hydrogen peroxide was quantified by the TiCl_4_ method (Jozef et al. 2009). Phosphate buffer (50 mM, pH: 6.5) was added into crushed culture. After centrifugation, 0.1 % titanium chloride in 20 % H_2_SO_4_ (1.5 mL) was added into supernatant (3 mL) and mixed thoroughly. After centrifugation at 15,000*g* at 4 °C for 20 min. Absorbance was spectrophotometrically measured at 410 nm. The amount of H_2_O_2_ was calculated from standardized curve (0.6–1.8 mM) H_2_O_2_ in buffer plus 0.5 mL of titanium chloride solution).

Superoxide anion was extracted by potassium phosphate buffer (pH 7.8) and estimated according to Sun and Hu (2005) by monitoring at 530 nm using NaNO_3_ as standard. Phosphate buffer (65 mM, pH: 7.8) were added into crushed algae solution and then centrifuged. Reaction mixture contained 2 mL of supernatant, 1.5 mL of phosphate buffer, 0.5 mL of hydroxylamine hydrochloride, after mixing, bathed at 25 °C water for 20 min, took 2 mL reaction solution, added 2 mL of sulfanilic and 2 mL of α-naphthylamine, bathed at 30 °C water for 30 min, and measured at 530 nm. Each treatment was replicated three times.

### Determination of enzyme activity

Peroxidase (POD) activity was measured according to guaiacol oxidation method (Gao 2005). Each sample had divided into the measuring tube and the blank tube, added with enzyme solution, 0.1 % guaiacol, distilled water, 0.18 % H_2_O_2_ (blank tube was not added), accurately react 10 min under 25 °C, 5 % metaphosphoric acid was added to terminate the reaction, measuring the absorbance under 470 nm.

Superoxide dismutase (SOD) activity was determined by tetrazolium reduction method (Gao 2005). One SOD unit was defined as the amount of enzyme required for inhibit 50 % of NBT photoreduction. Each sample was divided into three tubes, the measuring tube, the light control tube and the dark control tube respectively. Each tube was added with 550 mmol L^−1^ potassium phosphate buffer (pH 7.8), 130 mmol L^−1^ methionine solution, 750 μmol L^−1^ NBT solution, 20 μmol L^−1^ riboflavin solution, 100 μmol L^−1^ EDTA-Na_2_, distilled water, and the enzyme solution was added to the measuring tube, the same amount of distilled water was added to the other tubes. Then the tubes were placed under 1000Lx Fluorescent color reaction 15 min, covered with a black cloth to termination the reactions, make the dark control tube as a blank control and measured the absorbance at 560 nm.

Catalase (CAT) activity was determined using UV absorption method (Gao 2005), to reduce 0.1 within 1 min under A 240 was taken as an enzyme activity unit(U). Each sample had two tubes, added with Tris-HCl buffer (pH 7.0), distilled water, one tub was added with live enzymes, another was dead enzyme, preheat 3 min using a water bath at 25 °C, adding 200 mmol L^−1^ H_2_O_2_ and measuring the absorbance under A240 (distilled water zero) immediately.

Glutathione reductase (GR) activity was determined using the method of Schaedle (1977). To decreases 0.1 at A340 per milligram per minute was taken as an enzyme activity. GR catalyze following reaction: GSSH + NADPH → GSH + NADP^+^. GR activity was determined by measuring the change of NADPH. 1 mL reaction mixture containing 50 mmol L^−1^ potassium phosphate buffer (pH 7.8), 20 mmol L^−1^ EDTA, 1.5 mM NADPH, 5 mM GSSG, 200 μL enzyme solution, and measured the change of OD340 in 1 min under 20 °C immediately (extinction coefficient is 6.2 mmol L^−1^ cm^−1^).

All determinations were made in triplicate and data are expressed as means ± the standard deviation (SD). Statistical tests were carried out using the software SPASS ver. Differences between individual means were determined by Tukey’s post hoc multiple range test p < 0.05 for this procedure.

## Results

### Dose-effect of cadmium in *C. vulgaris* growth and composition of pigments

**Fig. 1:**
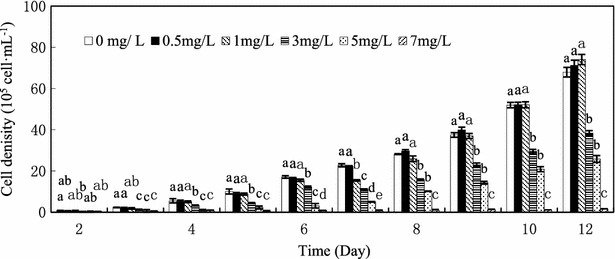
Cells density (10^5^ cells mL^−1^) of *C. vulgaris* under various Cd(II) concentrations. *Different lowercase letter* (*a*–*e*) indicate significant differences between treatments exposed to increasing metal concentration (p < 0.05). Values in *columns* followed by the *same letter*(s) are not significantly different according to Tukey’s test (p < 0.05)

Growth measured as cell density (Fig. 1). There were significant differences in the cell density of *C. vulgaris* under high Cd(II) treatments and cell density decreased in response to increasing cadmium doses, as shown in Fig. 1. The inhibited growth was mainly occurred under high cadmium concentration (3 mg L^−1^, 5 mg L^−1^), *C. vulgaris* could not be survived in 7 mg L^−1^ cadmium concentration. But under low cadmium supplements, there was barely no inhibited even slightly promotion after 12 days, and the effect was not obvious. The result indicted that *C*. *vulgaris* can be well tolerated with 1–5 mg L^−1^ cadmium, although the growth is inhibited under high concentration, *C*. *vulgaris* still can be lived in 5 mg L^−1^. Our research found that *C. vulgaris* has a high tolerance to cadmium.

**Fig. 2:**
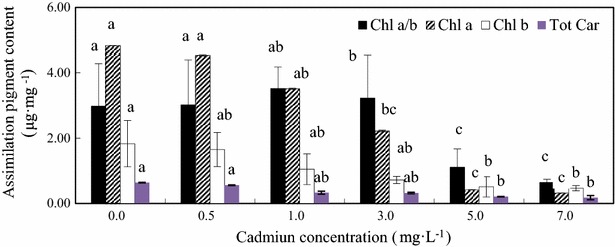
Assimilation pigment composition of *C. vulgaris* cultivated for 18 days in Cd(II) medium. *Different lowercase letter* (*a*–*c*) indicate significant differences between treatments exposed to increasing metal concentration (p < 0.05). Values in *columns* followed by the *same letter*(s) are not significantly different according to Tukey’s test (p < 0.05)

Effects of cadmium stress on *Chl a*, *Chl b* and total carotenoids of *C. vulgaris* are presented in Fig. 2. Cadmium had an adverse influence on *Chl a* production by *C. vulgaris*. The *Chl a* content significantly decreased (p < 0.05) from 4.83 to 0.32 µg mg^−1^, while the Cd(II) concentration was increased from 0 (control) to 7 mg L^−1^. A similar tendency was observed for *Chl b* upon cadmium exposure (Fig. 2). The total carotene production by *C. vulgaris* varied from 0.64 to 0.18 µg mg^−1^ for the control and 7 mg L^−1^ Cd(II), respectively. Cd(II) had an adverse effect (p < 0.05) on the total carotene production. The decrease of 54.04 and 93.37 % in *Chl a*, 60.65 and 74.32 % in *Chl b*, 50.00 and 71.88 % in total carotenoids was noticed following the treatment with 3 and 7 mg L^−1^ cadmium doses compared with control treatment, respectively. Cadmium treatments caused a significant change in the physiological competence (calculated as chlorophyll *a*/*b*) which increased with increasing Cd(II) doses up to 1 mg L^−1^ but decreased at 3 mg L^−1^. *Chl a* to *Chl b* ratios revealed that the damaging effect was found to be greater (by 42.6 %) on *Chl b* at 1 mg L^−1^ Cd(II) concentration while *Chl a* was affected more (by 93.4 %) under 7 mg L^−1^ Cd(II) concentration.

### The effect of cadmium on soluble protein content, hydrogen peroxide and superoxide anion content of *C. vulgaris*

**Fig. 3:**
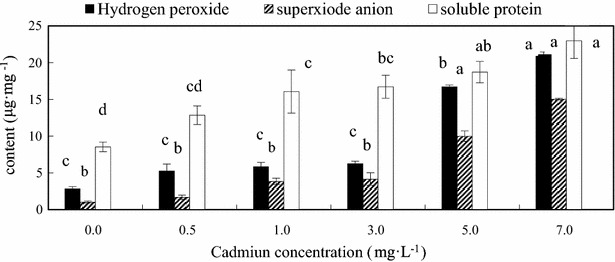
Contents of soluble protein, hydrogen peroxide and superoxide anion of *C. vulgaris* cultivated 18 days. *Different lowercase letter* (*a*–*c*) indicate significant differences between treatments exposed to increasing metal concentration (p < 0.05). Values in *columns* followed by the *same letter*(s) are not significantly different according to Tukey’s test (p < 0.05)

The influence of Cd(II) stress on protein, hydrogen peroxide and superoxide anion contents of *C. vulgaris* is given in Fig. 3. After 18 days exposure, effect of different Cd(II) concentrations on soluble protein content was significantly altered. Cd(II) stress had a significantly increasing trend on the protein content of *C. vulgaris* under the concentration 0–7 mg L^−1^ (Fig. 3).The effect of Cd(II) on hydrogen peroxide and superoxide anion were both significant (p < 0.001) (Fig. 3). Levels of hydrogen peroxide and superoxide anion elevated with increasing Cd content in the medium, significantly under the high concentration of 5 and 7 mg L^−1^, the hydrogen peroxide contents were increased by 5.90 times and 7.45 times, and superoxide anion were increased by 9.70 times and 14.59 times at 5 and 7 mg L^−1^ Cd(II) concentration, respectively. The highest contents of these ROS were observed in *C. vulgaris* cells treated with 7 mg L^−1^ Cd(II) concentration (7.45 times increase in hydrogen peroxide content, and 14.59 times increase in superoxide anion content) in the 18 days of cultivation.

### Activity of antioxidative enzymes

**Fig. 4:**
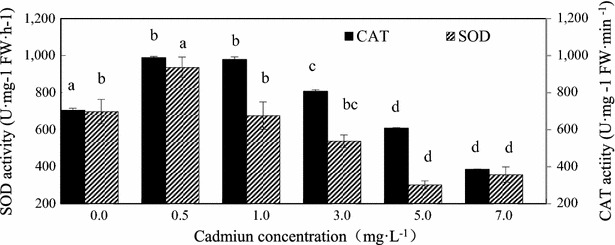
Contents of SOD and CAT of *C. vulgaris* cultivated 18 days *C. vulgaris* in Cd(II) medium. *Different lowercase letter* (*a*–*e*) indicate significant differences between treatments exposed to increasing metal concentration (p < 0.05). Values in *columns* followed by the *same letter*(s) are not significantly different according to Tukey’s test (p < 0.05)

**Fig. 5:**
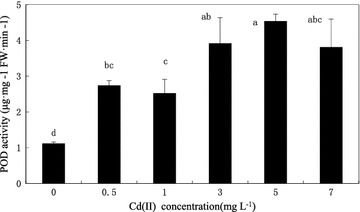
Contents POD of *C. vulgaris* cultivated 18 days in cadmium in Cd(II) medium. *Different lowercase letter* (*a*–*e*) indicate significant differences between treatments exposed to increasing metal concentration (p < 0.05). Values in *columns* followed by the *same letter*(s) are not significantly different according to Tukey’s test (p < 0.05)

**Fig. 6:**
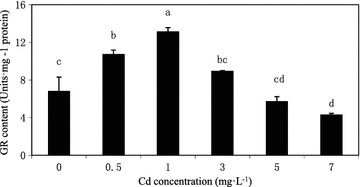
Contents GR of *C. vulgaris* cultivated 18 days in cadmium in Cd(II) medium. *Different lowercase letter* (*a*–*e*) indicate significant differences between treatments exposed to increasing metal concentration (p < 0.05). Values in *columns* followed by the *same letter*(s) are not significantly different according to Tukey’s test (p < 0.05)

Antioxidant biomarkers, Cd had significant effects on the activities of antioxidant enzymes at most of the experimental doses in comparison with the controls. Cadmium influenced the activity of antioxidant enzymes (SOD, POD, CAT and GR) involved in the scavenging of ROS (Figs. 4, 5, 6). Effect of different concentrations of cadmium on four enzymes was significant. The contents of SOD, CAT, GR were all first increases and then decrease with the cadmium concentration increasing, SOD and CAT content reached a maximum at 0.5 mg L^−1^ (Fig. 4), GR content reached a maximum at 1 mg L^−1^ (Fig. 6). The highest enhancement of the activity of antioxidant enzymes (34.18 % SOD, 38.79 % CAT) appeared as a consequence of algal exposure to 0.5 mg L^−1^ cadmium after 18 days of cultivation. Cadmium applied 1 mg L^−1^ stimulated the activity of GR by 92.38 %. The content of POD increased with the increasing cadmium concentration, and had slightly decreased at the concentration of 7 mg L^−1^, but was still higher than control group. The increase in POD level by 1.45 times, 1.26 times, 2.50 times, 3.06 times and 2.40 times was obtained in the culture growing in the presence of 0.5, 1, 3, 5, and 7 mg L^−1^ cadmium, respectively after 18 days of cultivation (Fig. 5). Results indicated that, 0.5 mg L^−1^ cadmium stimulated SOD by 34.18 %, CAT by 38.79 %, 1 mg L^−1^ cadmium enhanced the activity of GR by 92.38 %, whereas cadmium increased the activity of POD after 18 days of cultivation. And showed that cadmium stress at high concentration mainly POD works in *C*. *vulgaris*.

## Discussion

Decrease of the growth can be relatively easily determined and reflects physiological status of the algal cells (Juraj et al. 2011). Heavy metals had adverse effects on the growth of *Scenedesmus quadricauda* (Mohammed and Markert 2006; Stork et al. 2013) and *Spirogyra setiformis* (Çelekli et al. 2016) in cultures, same result also was found in this study, the inhibited of growth is mainly under high cadmium concentration, the growth of *C. vulgaris* decreased with the increasing cadmium concentration.

Pigment reduction was reported for *S. quadricauda* exposed to Cu (Kováčik et al. 2010) *C. pyrenoidosa* with perfluorooctanoic acid exposure (Xu et al. 2013), *S. obliquus* exposed to Cu (Chen et al. 2012) and carbamazepine (Zhang et al. 2012), and *C. vulgaris* exposed to Cr (Rai et al. 2013) and dichloromethane and dichloroethane (Wu et al. 2014). In the present study, Cd(II) stress could damage the biosynthesis of chlorophyll in *C. vulgaris*, which is in agreement with results of Küpper et al. (2003), Rai et al. (2013), and Çelekli et al. (2016). The increase of Chlorophyll *a*/*b* suggesting that the *Chl b* is more sensitive than *Chl b* under 0.5 mg L^−1^, the decrease suggesting that cadmium can cause some *Chl a* to be converted to *Chl b* by oxidation of the methyl group on the ring II (Chettri et al. 1998).

The Carotenoid also decreased, Cd(II) ions had an adverse effect on the total carotene content. An adverse effect was previously reported for the carotene production by *Ulva prolifera*, *U. linza* (Jiang et al. 2013) and *S. setiformis* (Çelekli et al. 2016) under Cd(II) exposure.

The soluble protein content increased under Cd(II) stress. The increase of soluble protein is because of soluble protein is related to a variety of metabolic processes in cells, heavy metal stress can induce related stress protein gene expression, which is a defense mechanism of plants to environment stress (Xu et al. 2007).

Cd(II) stress enhanced the accumulation of hydrogen peroxide and superoxide anion in *C. vulgaris*. Increasing hydrogen peroxide and superoxide anion content of algae due to exposure of heavy metal has been previously studied by many researchers (Wu et al. 2014; Çelekli et al. 2016). One of the mechanisms that was involved in the prevention of metal-induced cell destruction has been the synthesis of antioxidative enzymes (Wu and Lee 2008). Elevated levels of antioxidant enzymes SOD, CAT, GR and POD in *C*. *vulgaris* following the Cd treatment in this study indicated that these enzymes could act in combination to reduce the impact of metal toxicity, the same results has reported in *Acanthophora spicifera*, *Chaetomorpha antennina*, and *Ulva reticulate* (Babu et al. 2014).

A concentration-dependent increase in antioxidant activity was observed in the present work, similar to the reported results in *C. vulgaris* (Bajguz 2010). Increased glutathione levels had been shown to correlate with plant adaptation to extreme metal stress, and decreased glutathione pool shows marked alterations in response to metal stress (Jin et al. 2008; Masood et al. 2012). Therefore, the increased glutathione level noted in *C. vulgaris* when treated with Cd under 0–1 mg L^−1^, that may precede phytochelatin accumulation by intracellular sequestration of metal ions (De Vos et al. 1992). And the decreased glutathione level showed in *C. vulgaris* when treated with Cd under 3–7 mg L^−1^, similarly, exposure of Cd decreased glutathione of *A. spicifera* and exposure of Cu decreased glutathione of *C. antennina* (Babu et al. 2014).

Induction of SOD activity in plant cells had been correlated with increased tolerance to a variety of chemical compounds and physical stresses (Mittler 2002). Induced SOD activity can either be due to the increased production of ROS or the protective measure adopted by macroalgae against oxidative damage. CAT is one of the key enzymes involved in the removal of toxic peroxides as it quenches H_2_O_2_ to water and molecular oxygen. In the present study, the increase in CAT activity can be considered as an adaptive mechanism developed by plants (Reddy et al. 2005). Reduction of CAT activity observed at the higher concentration of metals might be attributed to inactivation of enzyme by ROS, decrease in synthesis of enzyme, or change in the assembly of its subunits (Verma and Dubey 2003). POD also plays an important role in respiratory metabolism in plants. In the present study, the activity of POD increases with the increasing cadmium concentration until 5 mg L^−1^ and then begins to decrease.

## Conclusion

Pollution in aquatic environments by metals has received considerable attention. Pollution of aquatic environments with heavy metals from natural water is a serious problem because of the toxicity of heavy metals to humans, fish and other live organisms. For this reason, pollution impact studies that focus on the aquatic environment are receiving more attention. This study confirmed that *C*. *vulgaris* showed a remarkable response to Cd(II) stress. Cadmium stress caused a variety of toxicity to *C*. *vulgaris*, such as algal biomass, chlorophyll, protein content decreased, H_2_O_2_ and O_2_^·−^ content increased. But *C*. *vulgaris* can adapt and regulation by changing the activity of SOD, POD, CAT and GR when subjected to cadmium stress in environment, thereby increasing the resistance to cadmium stress of *C*. *vulgaris.* Our research found that *C. vulgaris* has a high tolerance to cadmium. Hence, regulatory measures have to be taken by the authorities to limit the concentration of metal pollutants in the aquatic environment. This study also shows a range of physiological responses measured in this green alga under Cd(II) stress could be used as natural biomarkers or bioindicators of Cd contaminations in contaminated aquatic ecosystems.

## References

[CR1] Babu MY, Palanikumar L, Nagarani N, Devi VJ, Kumar SR, Ramakritinan CM, Kumaraguru AK (2014). Cadmium and copper toxicity in three marine macroalgae: evaluation of the biochemical responses and DNA damage. Environ Sci Pollut Res.

[CR2] Bajguz A (2010). An enhancing effect of exogenous brassinolide on the growth and antioxidant activity in *Chlorella vulgaris* cultures under metals stress. Environ Exp Bot.

[CR3] Bouzon ZL, Schmidt EC, de Almeida AC, Yokoya NS, de Oliveira MC, Chow F (2011). Cytochemical characterization and ultrastructural organization in calluses of the agarophyte *Gracilariopsis tenuifrons* (Gracilariales, Rhodophyta). Micron.

[CR4] Bradford MM (1976). A rapid and sensitive method for the quantity of protein dye binding. Anal Biochem.

[CR5] Çelekli A, Gültekin E, Bozkurt H (2016). Morphological and biochemical responses of *Spirogyra setiformis*, exposed to cadmium. CLEAN Soil Air Water.

[CR6] Chen H, Chen J, Guo Y, Wen Y, Liu J, Liu W (2012). Evaluation of the role of the glutathione redox cycle in Cu(II) toxicity to green algae by a chiral perturbation approach. Aquat Toxicol.

[CR7] Chettri MK, Cook CM, Vardaka E, SawidisT Lanaras T (1998). The effect of Cu, Zn and Pb on the chlorophyll content of the lichen *Cladonia convoluta* and *Cladonia rangiformis*. Environ Exp Bot.

[CR8] Dai HP (2012). Unraveling the mechanisms of cadmium tolerance and detoxification in *Populus* × *Canescens*.

[CR9] De Vos RCH, Vonk MJ, Vooijs R, Schat H (1992). Glutathione depletion due to copper induced phytochelatin synthesis causes oxidative stress in *Silene cucubalus*. Plant Physiol.

[CR10] Gao JF (2005). Plant physiology experimental guidance.

[CR11] Gülay BM, Yakup A (2011). Preparation of a composite biosorbent using *Scenedesmus quadricauda* biomass and alginate/polyvinyl alcohol for removal of Cu(II) and Cd(II) ions: isotherms, kinetics, and thermodynamic studies. Water Air Soil Pollut.

[CR12] Jiang HP, Gao BB, Li WH, Zhu M, Zheng CF, Zheng QS, Wang CH (2013). Physiological and biochemical responses of *Ulva prolifera* and *Ulva linza* to cadmium stress. Sci World J.

[CR13] Jin X, Yang X, Islam E, Liu D, Mahmood Q (2008). Effect of cadmium on ultrastructure and antioxidative defense system in hyperaccumulator and non-hyperaccumulator ecotypes of *Sedum alfredii* Hance. J Hazard Mater.

[CR14] Jozef K, Martin B (2007). Changes of phenolic metabolism and oxidative status in nitrogen-deficient *Matricaria chamomilla* plants. Plant Soil.

[CR15] Jozef K, Boǐivoj K, Jana K, Martin B (2009). Physiology of *Matricaria chamomilla* exposed to nickel excess. Ecotoxicol Environ Saf.

[CR16] Juraj P, Eliza S, Jana K, Tatiana K, Martin B (2011). Influence of long-term exposure to copper on the lichen photobiont *Trebouxia erici* and the free-living algae *Scenedesmus quadricauda*. Plant Growth Regul.

[CR17] Kováčik J, Klejdus B, Hedbavny J, Bačkor M (2010). Effect of copper and salicylic acid on phenolic metabolites and free amino acids in *Scenedesmus quadricauda* (Chlorophyceae). Plant Sci.

[CR18] Küpper H, Šetík I, Šetliková E, Ferimazova N, Spiller M, Küpper FC (2003). Copper-induced inhibition of photosynthesis: limiting steps of in vivo copper chlorophyll formation in *Scenedesmus quadricauda*. Funct Plant Biol.

[CR19] Liu H, Li L, Yin C, Shan B (2008). Fraction distribution and risk assessment of heavy metals in sediments of Mushui Lake. J Environ Sci.

[CR20] Lundholm N, Hansen PJ, Kotaki Y (2004). Effect of pH on growth and domoic acid production by potentially toxic diatoms of the genera *Pseudo*-*nitzschia* and *Nitzschia*. Mar Ecol Prog Ser.

[CR21] Luoma SN, Rainbow PS (2005). Why is metal bioaccumulation so variable? Biodynamics as a unifying concept. Env Sci Technol.

[CR22] Masood A, Iqbal N, Khan NA (2012). Role of ethylene in alleviation of cadmium-induced photosynthetic capacity inhibition by sulphur in mustard. Plant Cell Environ.

[CR23] Mittler R (2002). Oxidative stress, antioxidants and stress tolerance. Trends Plant Sci.

[CR24] Mohammed M, Markert B (2006). Toxicity of heavy metals on *Scenedesmus quadricauda* (Turp.) de brebisson in batch cultures. Environ Sci Pollut Res.

[CR25] Rai UN, Singh NK, Upadhyay AK, Verma S (2013). Chromate tolerance and accumulation in *Chlorella vulgaris* L.: role of antioxidant enzymes and biochemical changes in detoxification of metals. Bioresour Technol.

[CR26] Reddy MK, Alexander-Lindo RL, Nair MG (2005). Relative inhibition of lipid peroxidation, cyclooxygenase enzymes and human tumor cell proliferation by natural food colors. J Agric Food Chem.

[CR27] Romanowska E, Wróblewska B, Drożak A, Zienkiewicz M, Siedlecka M (2008). Effect of pb ions on superoxide dismutase and catalase activities in leaves of pea plants grown in high and low irradiance. Biol Plant.

[CR28] Schaedle M (1977). Chloroplast glutathione reductase. Plant Physiol.

[CR29] Schmidt ÉC, Nunes BG, Maraschin M, Bouzon ZL (2011). Effect of ultraviolet-B radiation on growth, photosynthetic pigments, and cell biology of *Kappaphycus alvarezii* (Rhodophyta, Gigartinales) macroalgae brown strain. Photosynthetica.

[CR30] Stanier RV, Kunisawa R, Mandel M, Cohen-Bazire G (1971). Purification and properties of unicellular blue–green algae (order: *Chrococcales*). Bacteriol Rev.

[CR31] Stork F, Backor M, Klejdus B, Hedbavny J, Kovacik J (2013). Changes of metal-induced toxicity by H_2_O_2_/NO modulators in *Scenedesmus quadricauda* (Chlorophyceae). Environ Sci Pollut Res.

[CR32] Sun Q, Hu JJ (2005) Plant physiology research technology. China, Northwest Agriculture and Forestry University Press, Shaanxi, pp 174–176

[CR33] Verma S, Dubey RS (2003). Lead toxicity induces lipid peroxidation and alters the activities of antioxidant enzymes in growing rice plants. Plant Sci.

[CR34] Wellburn AR (1994). The spectral determination of chlorophylls a and b, as well as total carotenoids, using various solvents with spectrophotometers of different resolution. J Plant Physiol.

[CR35] Wu TM, Lee TM (2008). Regulation of activity and gene expression of antioxidant enzymes in *Ulva fasciata* Delile (Ulvales, Chlorophyta) in response to excess copper. Phycologia.

[CR36] Wu S, Zhang H, Yu X, Qiu L (2014). Toxicological responses of *Chlorella vulgaris* to dichloromethane and dichloroethane. Environ Eng Sci.

[CR37] Xu QJ, Jin XC, Wang XM (2007). Effects of different concentration ammonium-n on *Hydrilla verticillata* antioxidant enzymes under Cd stress. Chin J Appl Ecol.

[CR38] Xu D, Li C, Chen H, Shao B (2013). Cellular response of freshwater green algae toperfluorooctanoic acid toxicity. Ecotoxicol Environ Saf.

[CR39] Zhang XL, Shi GX, Xu N, Zeng XM (2002). Effects of mercury and cadmium on some of physiological indicators of *Chara*. J Nanjing Norm Univ.

[CR40] Zhang PY, Yu J, Tang XX (2005). UV-B radiation suppresses the growth and antioxidant systems of two marine microalgae, *Platymonas subcordiformis* (Wille) Hazen and *Nitzschia closterium* (Ehrenb.) W. Sm. J Integr Plant Biol.

[CR41] Zhang W, Zhang M, Lin K, Sun W, Xiong B, Guo M, Cui X (2012). eco-toxicological effect of carbamazepine on *Scenedesmus obliquus* and *Chlorella pyrenoidosa*. Environ Toxicol Pharmacol.

[CR42] Zhou CF, Wu GR, Shi X (2001). The role of antioxidant system in Cu^2+^ stress resistance in *Alternanthera philoxeroides*. Acta Bot Sin.

